# Maternal and neonatal health expenditure in mumbai slums (India): A cross sectional study

**DOI:** 10.1186/1471-2458-11-150

**Published:** 2011-03-08

**Authors:** Jolene Skordis-Worrall, Noemi Pace, Ujwala Bapat, Sushmita Das, Neena S More, Wasundhara Joshi, Anni-Maria Pulkki-Brannstrom, David Osrin

**Affiliations:** 1 UCL Centre for International Health and Development, Institute of Child Health, 30 Guilford Street, London, WC1N 1EH, UK; 2 Department of Economics, Ca' Foscari University of Venice, Cannaregio, 873, 30121, Venezia, Italia; 3 Society for Nutrition, Education and Health Action (SNEHA), Urban Health Centre, Chota Sion Hospital, 60 Feet Road, Shahunagar, Dharavi, Mumbai 400017, Maharashtra, India

## Abstract

**Background:**

The cost of maternity care can be a barrier to access that may increase maternal and neonatal mortality risk. We analyzed spending on maternity care in urban slum communities in Mumbai to better understand the equity of spending and the impact of spending on household poverty.

**Methods:**

We used expenditure data for maternal and neonatal care, collected during post-partum interviews. Interviews were conducted in 2005-2006, with a sample of 1200 slum residents in Mumbai (India). We analysed expenditure by socio-economic status (SES), calculating a Kakwani Index for a range of spending categories. We also calculated catastrophic health spending both with and without adjustment for coping strategies. This identified the level of catastrophic payments incurred by a household and the prevalence of catastrophic payments in this population. The analysis also gave an understanding of the protection from medical poverty afforded by coping strategies (for example saving and borrowing).

**Results:**

A high proportion of respondents spent catastrophically on care. Lower SES was associated with a higher proportion of informal payments. Indirect health expenditure was found to be (weakly) regressive as the poorest were more likely to use wage income to meet health expenses, while the less poor were more likely to use savings. Overall, the incidence of catastrophic maternity expenditure was 41%, or 15% when controlling for coping strategies. We found no significant difference in the incidence of catastrophic spending across wealth quintiles, nor could we conclude that total expenditure is regressive.

**Conclusions:**

High expenditure as a proportion of household resources should alert policymakers to the burden of maternal spending in this context. Differences in informal payments, significantly regressive indirect spending and the use of savings versus wages to finance spending, all highlight the heavier burden borne by the most poor. If a policy objective is to increase institutional deliveries without forcing households deeper into poverty, these inequities will need to be addressed. Reducing out-of-pocket payments and better regulating informal payments should have direct benefits for the most poor. Alternatively, targeted schemes aimed at assisting the most poor in coping with maternal spending (including indirect spending) could reduce the household impact of high costs.

## Background

There have been numerous calls to improve neonatal survival and maternal health outcomes by stimulating demand for appropriate services [1-3]. Within this literature, the cost of care as a barrier to access has been frequently highlighted [4-6]. Similarly, the relationship between health spending and poverty has been discussed by Wagstaff [7], Van Doorslaer et al [8] and Whitehead et al [9,10], among others.

India accounts for more than 25% of maternal deaths globally [11]. The majority of studies on maternal health spending and service use in India have been conducted either at the macro-level (State or National) [6,12,13], or have had a predominantly rural focus (see for example [14]). Given that out of pocket payment is the principal method of financing health care throughout Asia [15], that over 72% of expenditure in India is financed out of pocket [13,16], and that Navaneetham et al [12] highlight significant difference in maternal care use between Indian States, there is a strong case for more focussed study of vulnerable populations.

Mumbai is India's most populous city, with more than 16 million people spanning multiple ethnic, cultural and linguistic groups. More than half of Mumbai's population currently live in slums [17], and families in these relatively deprived areas constitute the focus of this paper. Although no directly comparable study has yet been conducted within Mumbai's slum-dwelling population, a related study by Shah More et al [18] provides a comprehensive overview of maternal care seeking practices in Mumbai, with a focus on the poor. The authors describe high levels of antenatal care use (93%) and institutional delivery (90%), with antenatal care evenly split between public and private providers and delivery care slightly skewed towards the public sector. They describe the complexity of service choice in the Mumbai context and raise concerns about referral practices and the quality of care in both the public and private sectors [18]. By comparison, Griffiths and Stephenson conducted a qualitative study of care seeking in Mumbai and Pune [19]. They found that women perceived private services to be superior to government services, and poor perceptions of government care motivated home deliveries if women could not afford private care [19].

Within the context of calls to stimulate demand for care, poorer households may find it difficult to plan for and meet the costs of maternity care in a pluralistic health system. Inability to meet the cost of maternity care can act as a significant barrier to service access and may be a determinant of maternal and neonatal morbidity and mortality. Among those who do use services, health spending that constitutes a significant portion of household resources may also reduce other consumption, including spending on food and education, and may have both immediate and intergenerational effects on household poverty and the equity of health service delivery [7].

Health spending is generally considered 'catastrophic' when a household must reduce its basic expenditure to cope with health costs. This is assumed to happen when health expenditure exceeds a proportion (usually 40%) of total household income or expenditure, thus 'crowding out' other spending. Using a proportional measure such as this (health spending as a percentage of total spending), economists can approximate the welfare effects of medical expenditures [4,15,20-22]. Xu et al. [20] used data from 59 countries to explore variables associated with catastrophic health expenditure. They defined health expenditure as catastrophic if it exceeded 40% of income remaining after subsistence needs were met, and identified three key preconditions for catastrophic payments, all of which hold in the context of our study: availability of health services requiring payment, low capacity to pay, and a lack of prepaid health insurance. Su et al. [4] quantified the extent of catastrophic health spending and determined the risk factors in Nouna District, Burkina Faso. They used different thresholds of catastrophic health expenditure and found that even expenditure in the region of 6-15% of total income had catastrophic consequences. The key determinants of catastrophic expenditure were low economic status, modern medical care use (usually use of private services), illness episodes and a household member with chronic illness. A study in Thailand compared the incidence of catastrophic expenditures (>10% of total consumption, including food and non-food expenditure), before and after the introduction of universal health care coverage [22]. Households using private inpatient services were more likely to face catastrophic expenditure. Finally, van Doorslaer et al. [15] estimated the magnitude and distribution of catastrophic expenditure in 14 countries and territories, accounting for 81% of the Asian population. They found that countries relying most heavily on out-of-pocket (OOP) financing had the highest incidence of catastrophic payments.

In short, the literature consistently argues that out-of-pocket medical expenditure increases the risk of catastrophic spending and medical poverty. However, Flores et al. [23] argue that this definition of catastrophic spending is insensitive to how spending is financed through coping strategies. They provide an extensive review of the literature on coping with health payments, and consider not only the extent of health spending but also the strategies through which it is financed. Using nationally representative data from India, they found that coping strategies financed up to 75% of inpatient care. Capacity to draw on savings, assets, credit and transfers from friends and relatives may thus have protected the consumption of other goods, at least in the short term. They suggest that ignoring coping strategies may overstate the risk to short-run consumption, exaggerate the short-run scale of catastrophic payments, and potentially understate the long-run burden of health payments that may include significant debt financing.

Aside from measuring the impoverishing effect of health spending, the incidence of catastrophic payments is also an indicator of equity in health service provision. For example, Xu et al. [21] investigated whether the abolition of user fees levied at government health facilities in Uganda increased access for the poor and reduced the risk of catastrophic health expenditure. They defined expenditure as catastrophic if it exceeded 40% of income remaining after subsistence needs were met and found that utilization among the poor increased after the abolition of fees. Unexpectedly, however, the incidence of catastrophic spending among the poor did not fall. The authors argue that the most likely explanation was that frequent unavailability of drugs at government facilities after 2001 forced patients to purchase from private pharmacies. Moreover, informal payments may have risen to offset lost revenue from formal fees. This finding points to the importance of measuring both formal and informal health expenditures.

In addition to the formal and informal constituents, the price of health services usually incorporates both a direct and indirect cost. McIntyre et al. [24] and Russell [25] provide detailed overviews of health expenditure studies, identifying direct and indirect costs commonly used to quantify the economic consequences of ill health. Examples of direct medical costs commonly include scheduled user fees (or the price of traditional or private care), drug costs, informal payments and payment for diagnostic tests [26-29]. Indirect costs generally cover productivity losses and the opportunity cost of care seeking, including travel and waiting times [30-33].

While analysing spending can highlight the drivers of medical poverty and inequitable care, the literature on health equity is also concerned with the progressiveness of financing. The progressiveness of a financing system refers to "the extent to which payments for health care rise or fall as a proportion of a person's income as income rises. A progressive system is one in which health care payments rise as a proportion of income as income rises, whilst a regressive system is one in which payments fall as a proportion of income as income rises" [34]. There are different ways to capture progressiveness in health financing. The most direct is to examine health spending as a share of ability to pay. For example, a study from Sierra Leone showed that the rural poor can be disproportionately disadvantaged by user charges for health care, spending a higher percentage of their incomes on health care than wealthier households [35]. By comparison, Roy and Howard [36] examined how well the Indian healthcare system protected households of differing living standards against the financial consequences of health shocks. They found that OOP payments increase with ability to pay. They suggested two explanations for this observed relationship: firstly, a lack of insurance requiring the better-off to pay more to secure higher quality care, and secondly, the poor may limit expenditure more stringently and possibly forego care altogether. In this literature there is a gap in recent evidence as it relates to the urban poor in low and middle income settings.

In summary, equitable healthcare and the reduction of medical poverty are key goals of health systems and financing reform. In this paper, we analyzed direct and indirect expenditure on private and public maternity care in urban slum communities in Mumbai. The first objective was to understand what proportion of household spending was allocated to maternal health expenditure, and how this proportion varied by socioeconomic status (SES) as a measure of the progressiveness of payments. The second objective was to analyze the sources of finance (coping strategies) used to meet this expenditure. Finally, we analysed the relative impact of maternal health expenditure on current consumption, to estimate the incidence of catastrophic payments.

## Methods

### Data Collection

We used data on delivery care expenditures, sources of finance and socioeconomic characteristics, collected by the Society for Nutrition, Education and Health Action (SNEHA) as part of the City Initiative for Newborn Health. The City Initiative for Newborn Health is a collaboration of SNEHA, the Municipal Corporation of Greater Mumbai, the ICICI Centre for Child Health and Nutrition, and the UCL Centre for International Health and Development. Interviews were conducted between 2005 and 2006. The sample consisted of 1204 post-partum women living in 48 Mumbai slum communities. The study was conducted in 6 municipal wards, which defined the population for a larger cross-sectoral initiative [17,37]. Data collection and usage was approved by the Mumbai Independent Ethics Committee for Research on Human Subjects (IECRHS).

As part of the initiative, a vital registration system was set up to monitor all births, stillbirths, neonatal, infant and under-five deaths. Deaths of females in the age group 10-50 years were also recorded. The study was headed by a project coordinator (NSM) and data collection activities were managed by two project officers (UB and SD), each responsible for three municipal wards. Vital events were identified by 99 locally resident women, each covering an average 600 households. Remuneration was based on verified events. Births and deaths were communicated to one of 12 interviewers, each responsible for 4 clusters, who confirmed them by home visits and revisited for a postnatal interview at about six weeks after delivery. Interviewers had had higher secondary schooling, were trained for two weeks and met for feedback and ongoing training weekly. The interview - developed over multiple pilot iterations - was based on a predominantly closed questionnaire with questions on demography, socioeconomic factors, and maternity care.

Respondents gave verbal consent to interview and were assured of data confidentiality. Team members who encountered illness in mothers or infants had an ethical responsibility to recommend that they visit a health facility. Each completed interview was checked by the interviewer and by a supervisor. Supervisors visited homes to crosscheck every tenth interview, and observed interviews randomly. Every third completed questionnaire was checked by a project officer, who met with supervisors weekly to review progress. Questionnaires were all crosschecked at a central office and entered in a database in Microsoft Access with validation constraints and enforced referential integrity. The data management officer checked electronic data from every tenth questionnaire, and compared every fifteenth questionnaire entered against its original. Information provided by participants remained confidential, with access restricted to interviewers, supervisors, data auditors, officers, and analysts. Analyses and outputs were anonymized.

For three months from January to March 2007, we added to the basic data collection a questionnaire on maternal and newborn health care expenditure. All women who gave birth and received the standard interview completed this adjunct questionnaire. Recruitment was sequential until 200 questionnaires had been completed in each of six municipal wards. To account for the different recall heuristics that respondents might use in answering questions on expenditure, we provided two options: answers on the costs of individual items and answers on the total costs under each heading. For example, respondents who gave birth at home would more easily be able to recall expenditure on each item that they needed, including any fee levied by a traditional birth attendant. Alternatively, respondents who gave birth in hospital were usually presented with an aggregate bill for services.

The analysis was limited to households in which at least one of the members experienced a health event. This limitation increased confidence that observed differences in OOP payments are a reflection of the health system rather than differences in health status. Among others, Fabricant, Kamara and Mills [35], Roy and Howard [36], and Chaudhuri and Roy [38] applied this method.

### Data Analysis

Data analysis was performed using Stata, version 10. An asset-based index of socio-economic status was constructed using principal components analysis (PCA), a multivariate statistical technique used to describe SES differentiation within a population (for technical details on how the PCA was generated, see Vyas and Kumaranyake [39]). The procedure followed to construct the SES index can be summarised in three main steps: selection of the asset variables; application of PCA; and classification of households into socio-economic groups. When measuring SES, previous studies used variables such as literacy or education level, living in rented or owner-occupied housing, asset ownership, and dwelling and sanitation characteristics [40,41]. We carried out correlation analysis that allowed us to select the following determinants of wealth in this setting: maternal literacy, husband's education, electricity source, water source, house ownership, construction material of the home, type of toilet facility, holding a ration card, and ownership of the following assets: a pressure cooker, gas cylinder, chair, cot, table clock, radio, telephone, fridge or TV. The index was then constructed from the first component of the PCA to maximize scale reliability (0.81) and minimize skewness (-0.22) [39,42]. Each household in the dataset was assigned a wealth score or SES index value and, on the basis of that score, was allocated to a wealth quintile.

The progressiveness of health expenditure was measured by comparing average spending in the lowest and highest quintiles and testing for significant differences in the means using a t-test^1^. Significance was attributed at a 95% confidence level. If there was no significant difference in spending - if the lowest quintile did not spend significantly more - expenditure was defined as weakly regressive. If the lowest quintile spent more than the highest on any item, spending was defined as strongly regressive. This conservative measure of regressive spending was based on the assumption that individuals in the lowest quintile earned significantly less, and if they spent the same as (or more than) an individual in the highest quintile their expenditure would constitute a greater proportion of their income and would thus be regressive.

The equity of health spending was then further analysed using a Kakwani Index [43]. The Kakwani Index is one of the most widely used measures of equity in health care payments [44-46]. It is defined as twice the area between a payments' concentration curve and the Lorenz curve and is calculated as;(1)

Where:

C = the health payments' concentration index

G = the Gini coefficient of the SES score (our proxy of ability to pay).

While the Gini coefficient does not vary (*G *is constant in the calculation of the Kakwani index), we calculated different concentration indices (C) for all the categories of payments collected in the study (total, direct, indirect, public, private, etc). The value of πk ranges from -2 to 1. A negative number indicates regressive spending as the concentration curve lies inside the Lorenz curve, while a positive number indicates progressive spending as the concentration curve lies outside the Lorenz curve. In the case of proportionality, the index is zero (i.e. the concentration lies on the top of the Lorenz curve).

While the analysis of equity described above could be calculated using the SES index as a proxy of ability to pay, calculating catastrophic health spending required a measure of total income or expenditure. Household, rather than individual or extra-household, income/expenditure is the common measure of the financial resources available for health seeking [28,33,47]. As neither household income nor expenditure were collected within this study, we used national data [48] to impute per capita expenditure by housing type [23]. To this end, we used Monthly Per-capita Consumer Expenditure (MPCE) as published in the Indian National Sample Survey (2005-2006) [48], which provides per-capita expenditure by different categories. We chose the classification of 'per-capita expenditure by dwelling type' (pucca, semi-pucca, katcha) because this information perfectly matched the information on dwelling characteristics in our dataset, and because it provided an acceptable proxy of wealth^2^. We then multiplied monthly per-capita expenditure by twelve to obtain annual per-capita expenditure. We followed the example of Flores et al [23] and reported catastrophic spending both before and after adjusting for coping, by reporting total expenditure as a percentage of total income and then reducing total spending by the amount financed through coping and calculating the reduced expenditure as a proportion of what income would have been if medical expenditure had been zero [23,49]. Expenditure was adjusted for coping using the following formula as applied in Flores et al [23]:(2)

Where:

*P*_*i *_= the 'coping'-adjusted health expenditure ratio

*δ*_*i *_= maternity care expenditure

*x*_*i *_= total per-capita expenditure

*ϑ*_*i *_= amount of maternity care exp. financed with coping strategies

## Results and Discussion

### Health expenditure

*Table *1 shows the descriptive statistics for total expenditure on all maternity care as well as antenatal, delivery, postnatal and neonatal care. The large standard deviations should be noted as they indicate a significant level of dispersion in the data, which is the reason why median expenditure is also presented. Indian Rupees were converted to US Dollars using an inflation-adjusted nominal market-based 2010 exchange rate (USD 1 = 0.031002446 Rupee). Values are rounded to the nearest dollar. All the figures include direct and indirect expenditure. Spending on delivery care was the largest contributor to total spending.

**Table 1:** Total expenditures for antenatal, delivery, postnatal and neonatal care (in 2010 USD, inflation adjusted)

	**Total Expenditure**	**Total ANC Expenditure**	**Total Delivery Care Expenditure**	**Total PNC Expenditure**	**Total NNC Expenditure**
	
N	1204	1204	1204	1204	1204
Mean	271	74	138	26	33
Std Dev.	387	96	188	77	265
Median	174	50	68	10	4
Min	0	0	0	0	0
Max	6478	1625	1563	1602	6293

SD: Standard deviation

*Table *2 shows the descriptive statistics for direct expenditure as a proportion of total expenditure on all care as well as antenatal, delivery, postnatal and neonatal care. Total indirect expenditure as a proportion of total expenditure is the inverse of the figures presented. Indirect expenditure includes spending on transport, subsistence, loss of earnings for self and companion and tips to staff, which constituted respectively an average 35%, 25%, 23% and 17% of total indirect expenditure. Direct spending constituted the larger proportion of total expenditure.

**Table 2:** Direct expenditure as proportion of total expenditures

	**Direct expenditures as a proportion of**:				
	**Total expenditure**	**Antenatal care**	**Delivery care**	**Postnatal care**	**Neonatal care**
	
N	1203	1151	1197	788	736
Mean	81%	81%	74%	87%	87%
SD	16%	21%	27%	23%	26%
Median	86%	88%	83%	100%	100%
Min	6%	0	0	0	0
Max	100%	100%	100%	100%	100%

SD: Standard deviation

In summary, delivery expenditure represented the largest proportion of maternal spending, 51% of total expenditure. The figure for delivery expenditure presented in *Table *1 is an average of the cost of an institutional delivery and a home delivery. The average delivery cost for institutional births was $156 (SD. $197) and for home deliveries a significantly lower $30 (SD $32). Although direct spending was the largest contributor to all costs, indirect spending constituted 19% of total costs, rising to 26% for delivery costs. Indirect spending was thus a significant contributor to the cost of maternity care, suggesting that the removal of user fees alone will not remove the burden of maternity spending on the poor.

### Relative differences in expenditure

The aim of this section is to explore the relative impact of health spending on the poor. Of particular interest is whether the burden of spending was regressive. The progressiveness of spending is established by comparing spending between the first and fifth wealth quintiles. As this is a sample of slum dwellers it should be noted that the fifth quintile refers to the least poor and the first quintile to the most poor. The wealthy are not represented in this sample. Total indirect and total direct spending are analysed in turn. Spending in the private versus public sectors and home deliveries are then compared because of the pluralistic nature of the health setting.

**Figure 1:**
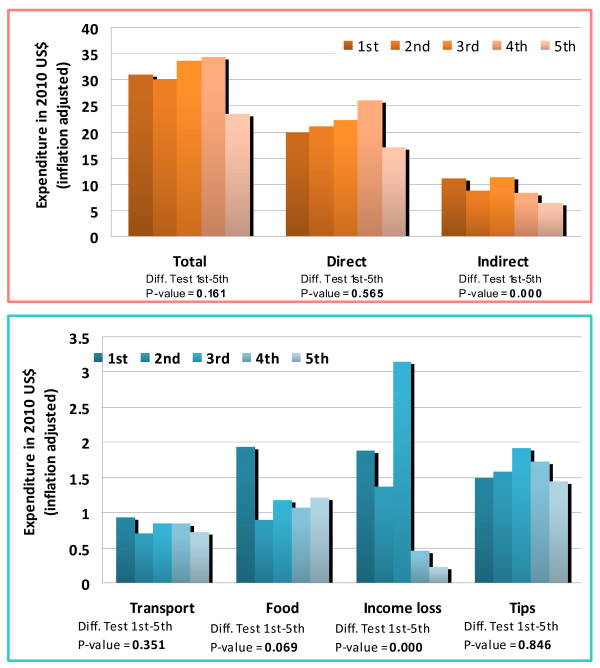
Spending by wealth quintile in the public sector

**Figure 2:**
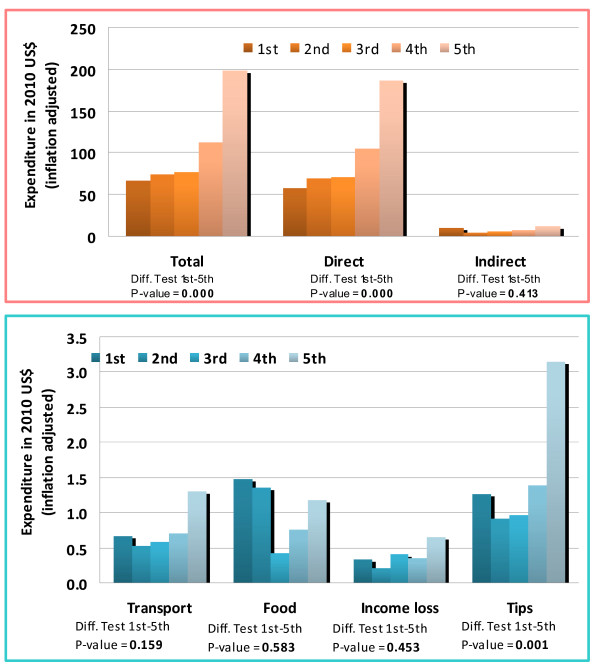
Spending by wealth quintile in the private sector

Total health expenditure was significantly higher in the highest quintile and we therefore cannot conclude that it was regressive based on this measure. However, total indirect expenditure was weakly regressive by this measure since there was no significant difference between spending in the lowest and highest quintiles. Although the highest wealth quintile spent significantly more on transport and tips, individuals in the lowest quintile experienced a significantly higher loss of income. Loss of income was therefore strongly regressive and spending in this category drove the weakly regressive burden of indirect health spending as a whole. The lowest quintile also spent significantly more indirectly as a proportion of total health expenditure. The importance of loss of income suggests potential policy solutions discussed later in the paper. These results are illustrated in Figures 1 and 2, where they are also disaggregated by sector.

Comparing spending between the public and private sectors, direct spending in the public sector was weakly regressive and indirect spending strongly regressive. Disaggregating indirect expenditures in the public sector, we found that tips, transport and food/subsistence were weakly regressive, while loss of income was again strongly regressive. By comparison, indirect spending in the private sector was weakly regressive while direct spending was higher in the highest quintile. We cannot conclude that private tips were regressive, although spending on transport and subsistence were weakly regressive when consulting a private provider. The loss of income was weakly rather than strongly regressive in this sector, perhaps reflecting shorter waiting times.

Finally, home deliveries were more likely in the lower quintiles, suggesting that they were an inferior good, i.e. as income increased less of the good was demanded. Similarly, public services were used more by lower quintiles, again suggesting that they were an inferior good. Demand for institutional deliveries and private services increased with wealth and as such were normal goods. The equity of health spending was also analysed using the Kakwani Index. *Table *3 shows this index across the full range of expenditure categories analysed in this paper. As explained before, a negative sign and a significant *p*-value indicate regressive spending, while a positive sign indicates progressive spending.

**Table 3:** Kakwani Index by type of out of pocket expenditure

	Kakwani Index	**St. Err**.	P-Value
Total Expenditure	0.013	0.024	0.594
Direct Expenditure	0.037	0.027	0.161
Indirect Expenditure	-0.152	0.020	0.000
Transport	-0.126	0.025	0.000
Food	-0.220	0.034	0.000
Income loss	-0.418	0.090	0.000
Tips	-0.090	0.025	0.000
Total Antenatal Exp.	-0.042	0.022	0.052
Direct Antenatal Exp.	-0.021	0.024	0.385
Indirect Antenatal Exp.	-0.156	0.030	0.000
Total Delivery Exp.	0.024	0.022	0.290
Direct Delivery Exp.	0.052	0.025	0.041
Indirect Delivery Exp.	-0.151	0.026	0.000
Total PNC Exp.	-0.048	0.050	0.333
Direct PNC Exp.	-0.046	0.053	0.388
Indirect PNC Exp.	-0.072	0.050	0.150
Total NNC Exp.	0.139	0.139	0.316
Direct NNC Exp.	0.167	0.148	0.260
Indirect NNC Exp.	-0.217	0.092	0.019
Total Public Exp. (delivery care)	-0.214	0.033	0.000
Direct Public Exp. (delivery care)	-0.185	0.043	0.000
Indirect Public Exp. (delivery care)	-0.284	0.038	0.000
Total Private Exp. (delivery care)	0.068	0.034	0.047
Direct Private Exp. (delivery care)	0.079	0.036	0.027
Indirect Private Exp. (delivery care)	-0.069	0.047	0.140

We find that total indirect expenditure was significantly regressive, as were all categories of indirect spending. Indirect antenatal expenditure was similarly regressive. Direct delivery expenditure was significantly progressive, while indirect delivery expenditure was regressive. Indirect spending on neonatal care was significantly regressive, as was all delivery care spending in the public sector (total, direct and indirect). Total and direct spending on delivery care in the private sector was progressive.

### Financing health expenditure

In this section we analysed the source of finance for maternal and neonatal health expenditures, comparing only the highest and lowest wealth quintiles. As *Table *4 illustrates, most maternal and neonatal expenditure was financed with savings (57.46%), current income from wage and salary (39.14%) and borrowing (17.41%).

**Table 4:** Proportion financing maternity expenditures by different sources

Variable	Proportion	95% confidence interval
Wage, salary	39.14%	[37.73%, 40.54%]
Cut food expenditures	0.17%	[0.05%, 0.28%]
Mortgage of jewelry, land, livestock	1.74%	[1.36%, 2.12%]
Borrowing	17.41%	[16.32%, 18.51%]
Sale of assets	0.17%	[0.05%, 0.28%]
Savings	57.46%	[56.04%, 58.89%]
Cut expenditure on education or health care	0.33%	[0.17%, 0.50%]
Other sources	0.66%	[0.43%, 0.90%]

As shown in *Table *5, women in the lowest quintile financed a significantly greater proportion of their total expenditure through wages. Flores et al [23] describe spending from wages as contributing primarily to transient rather than chronic poverty, whether or not spending levels are categorised as catastrophic. However, our finding in the previous section, that women in the lowest quintile had the greatest loss of wage income while seeking care, suggests that health spending placed a double burden on the poorest. In addition, we found that women in the lowest quintile funded significantly more spending from borrowing, a risk factor for chronic poverty [23]. Women in the highest quintile financed a greater proportion of their spending from savings, suggesting that they were better able to plan for these expenditures and to absorb health spending without necessarily contracting other consumption.

**Table 5:** Source of finance by wealth quintile

**Source of finance (% of total expenditure)**	**Wealth Quintile**					**P-value for mean difference test between lowest and highest quintiles**
	**Lowest**	**2nd**	**3rd**	**4th**	**Highest**	
		
*Wage and salary*	32	31	35	32	25	0.062
*Savings*	48	47	53	54	60	0.005
*Borrowing*	18	19	9	12	13	0.074
*Other sources*	1	3	2	3	2	0.349

### Catastrophic health spending in the study population

**Figure 3:**
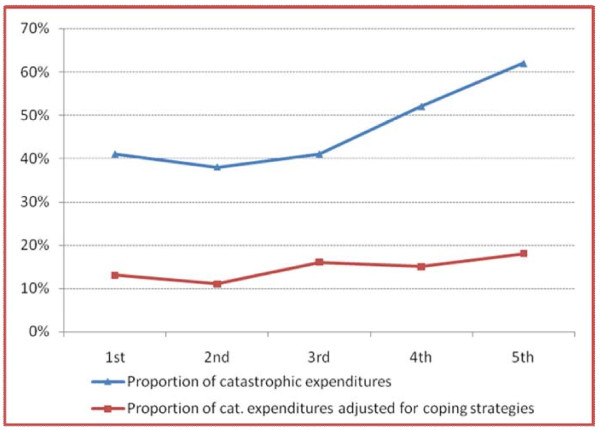
Catastrophic spending by wealth quintile

Since household income or expenditure was not collected in the study, we used national data [48] to impute expenditure by housing type [23]. As discussed earlier, health spending is commonly considered catastrophic if it exceeds 40% of total income/expenditure [50]. Using this definition, 41% of respondents spent catastrophically on maternal and neonatal care. A significantly higher proportion of catastrophic spending occurred in the highest quintile, possibly because women in the lowest quintile were forced to control spending by opting for inferior services (such as public providers and home deliveries) or by foregoing care altogether, a phenomenon also observed in other studies from this context and discussed further later [18,19]. We followed the example of Flores et al and adjusted for coping strategies [23]. After adjustment, 15% of the sample experienced catastrophic spending on maternal and neonatal health. There was no significant difference in the incidence of catastrophic spending across wealth quintiles. This result highlights that women in the highest quintile could afford to spend more, not only because they were less poor, but also because they had more access to coping strategies in the form of savings (Figure 3).

## Conclusion

We analyzed expenditure on maternal and neonatal care in urban slum communities in Mumbai. The first objective was to describe the constituent parts of maternal spending. We found that the cost of delivery care was the greatest maternal expense and that the cost of delivering in a facility was, on average, more than five times the cost of delivering at home. Formal and informal payments for delivery care constituted 90% and 10% of total delivery care expenditure, respectively. We also found that, while direct costs comprised the greatest share of maternal spending - indirect costs constituted a sizeable 19% of total maternal spending on average.

Our next objective was to understand the equity of maternal spending by measuring how spending varied by socioeconomic status (SES). We found that indirect spending was regressive for all types of care, in all sectors. Public services and home deliveries were inferior goods used by poorer families and as such, all spending in the public sector was regressive. As demand for the private sector increased with SES, total and direct spending on private maternal care had a positive Kakwani Index i.e. was progressive. Within indirect costs, those in the higher SES quintiles spent more on transport and tips while the poorest spent more on lost wage income. The poorest also faced a significantly higher proportion of informal payments. These findings, together with the observation that women in the lowest quintile funded significantly more spending from borrowing, highlighted how those in the lower quintiles are most at risk of both transient and chronic poverty [23].

The final objective was to analyze catastrophic spending and the sources of finance (coping strategies) used to fund maternal health expenditure. Most maternal and neonatal expenditure was financed with savings (57.46%), current income from wage and salary (39.14%) and borrowing (17.41%). There were, however, significant differences in coping strategies across the wealth quintiles, with the poorest relying more on wages and borrowing to finance spending and the least poor spending relatively more out of savings. Before adjusting for coping strategies, 41% of our sample spent catastrophically on maternal and neonatal care. A significantly higher proportion of catastrophic spending occurred among women in the highest quintile. This could be because women in the lowest quintile were forced to control spending by opting for cheaper inferior services (public sector care and home deliveries), less frequent consultations, or foregoing care altogether. Conversely, women in higher wealth groups - although not earning very much more given the nature of the sample - were more likely to use higher-cost private services. These results suggest that women in the highest quintile could afford to spend more, not only because they were less poor, but also because they had more access to savings. The findings reflect behaviour observed in at least two other studies conducted in Mumbai [18,19]. In short, poorer people have no safety net and lack flexibility and confidence in future income. The other issue highlighted by Shah More [18] is one of 'culture'. They found that urban communities are aspirational, and that one of the aspirations is to achieve 'modernity' in health care. Depending on socioeconomic status, maternity care steps up through a sequence from home delivery, via public sector antenatal care, to public sector delivery, to private sector delivery (the highest aspiration). Identification with a higher socioeconomic stratum is also aspirational and may lead to uptake of health care services that proves more catastrophic than anticipated [18].

As with most analyses of this kind, the study has a number of limitations. The extent to which the findings can be generalised to greater Mumbai or to slum-dwellers in other cities, would depend on how comparable is the supply of maternal health services among other factors. The study also has little to say about the quality of care received and whether services perceived to be inferior were in fact of lower quality. Finally we should be circumspect about the results of the catastrophic spending analysis as the data on household income were imputed from a national dataset and not collected within the primary survey. That said, the incidence of catastrophic spending in this setting was so high that even quite large changes in the exact measure would not change the fact that a significant percentage of households were being impoverished by maternal health spending. After adjusting for coping, 15% of our sample experienced catastrophic spending. There was no significant difference in the incidence of catastrophic spending across quintiles after adjusting for coping. A final limitation of the study is that respondents, in the case of direct spending only, were allowed the option of reporting total rather than disaggregated spending. These responses may be vulnerable to a downward bias [51-53].

These results highlight the significant problem of inequitable and catastrophic health spending in this urban population. However, they also suggest some potential policy solutions. Given high, and highly regressive, indirect spending on care, the removal of user fees alone is unlikely to relieve the burden on the poorest. Cash transfers (conditional or unconditional) such as the Janani Suraksha Yojana (JSY) scheme launched by the Government of India may be effective in urban Mumbai if it is possible to better target poorer mothers [54]. This may go some way towards reducing the negative effects of OOP spending among slum dwellers and may compensate partially for the loss of earnings amongst the poorest. In addition, better regulation of informal payments, with the aim of their prevention or removal, would further reduce the burden on the poorest. For the small number in formal employment, requiring employers to allow pregnant employees to attend maternal care services without loss of earnings should reduce the double burden on that group. Given that the poorest are more likely to deliver at home or to use public care, policy makers should also ensure that these care options provide an acceptable level of care for mothers and their neonates. Other strategies to reduce loss of earnings and the 'double hit' on wages experienced by the most poor would further improve the equity of, and access to, maternal and neonatal care in urban Mumbai. Without changes such as these, the poorest will continue to forego higher-cost institutional deliveries, placing themselves and their neonates at risk.

## Competing interests

The authors declare that they have no competing interests.

## Authors' contributions

JSW designed and supervised the data analysis and participated in the drafting of the manuscript. NP conducted the analysis and participated in the drafting of the manuscript. UB, SD, NSM, WJ and DO conceived the study, designed the data collection procedure and oversaw data collection and entry. AMPB contributed to the drafting of the final manuscripts and assisted with the data analysis. All authors participated in drafting the manuscript and have read and approval the final manuscript.

## Endnotes

1. We used the Stata command "ttest" applying the "by(groupvar)" option that specifies the variable that defines the two groups that ttest will use to test the hypothesis that their means are equal. In our study, the two groups are the lowest and the highest quintiles.

2. Data on Monthly Per-capita Consumption Expenditure (MPCE) by dwelling type is taken from Table 11U in the Appendix A of 48. National Sample Survey Organisation: Household Consumer Expenditure in India 2005-2006. In., vol. 523: NSSO; 2006. The average MPCE is 686 Rs, 822 Rs, 1431 Rs for individuals living respectively in Katcha, Semi-pucca and Pucca.

## Pre-publication history

The pre-publication history for this paper can be accessed here:

http://www.biomedcentral.com/1471-2458/11/150/prepub
